# First person – Imad Soukar

**DOI:** 10.1242/bio.060190

**Published:** 2023-11-10

## Abstract

First Person is a series of interviews with the first authors of a selection of papers published in Biology Open, helping researchers promote themselves alongside their papers. Imad Soukar is first author on ‘ Analysis of the chromatin landscape and RNA polymerase II binding at SIN3-regulated genes’, published in BiO. Imad conducted the research described in this article while a PhD student in Lori A. Pile's lab at Wayne State University, Detroit. He is now a Postdoc in the lab of Rhoda Alani at Boston University, Boston, investigating manipulation of epigenetic control of genes, to solve important scientific problems.


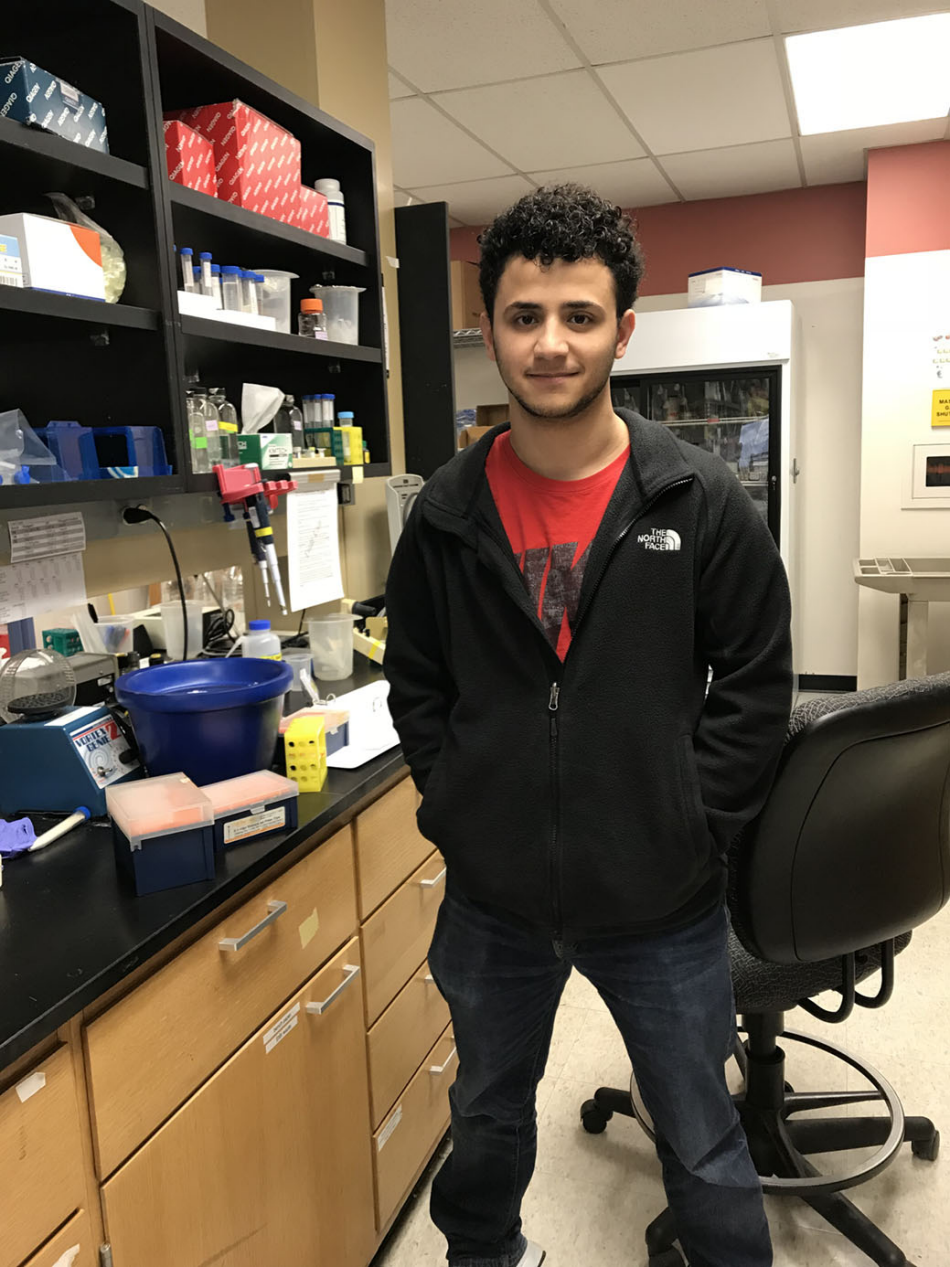


**Imad Soukar**

**Describe your scientific journey and your current research focus**

My family immigrated to the US from Syria in 2007. I was a very good student in Syria, consistently the top of my class. In the US I was not a good student. Learning a new language and culture was difficult, and my studies suffered. In my junior year of my undergraduate studies, I met my PhD advisor, and I was immediately hooked on science. I enjoyed her teaching of molecular biology and decided to volunteer as an undergraduate researcher in her lab for the summer. I enjoyed my time doing research that summer and decided to pursue a PhD in molecular biology in her lab. I am currently a post-doctoral researcher in the Alani group at Boston University.

**Who or what inspired you to become a scientist?**

My PhD advisor, Lori Pile, whom I met when she taught the undergraduate course molecular biology. Her teaching style and love for science inspired me to become a scientist.

**How would you explain the main finding of your paper?**

The main finding of the paper is the potential mechanism of how genes are regulated by an important protein called SIN3.

The main finding of the paper is the potential mechanism of how genes are regulated by an important protein called SIN3.

**What are the potential implications of this finding for your field of research?**

These findings will aid in future hypothesis forming and experiments. We laid out the potential mechanism of how SIN3 works.

**Figure:**
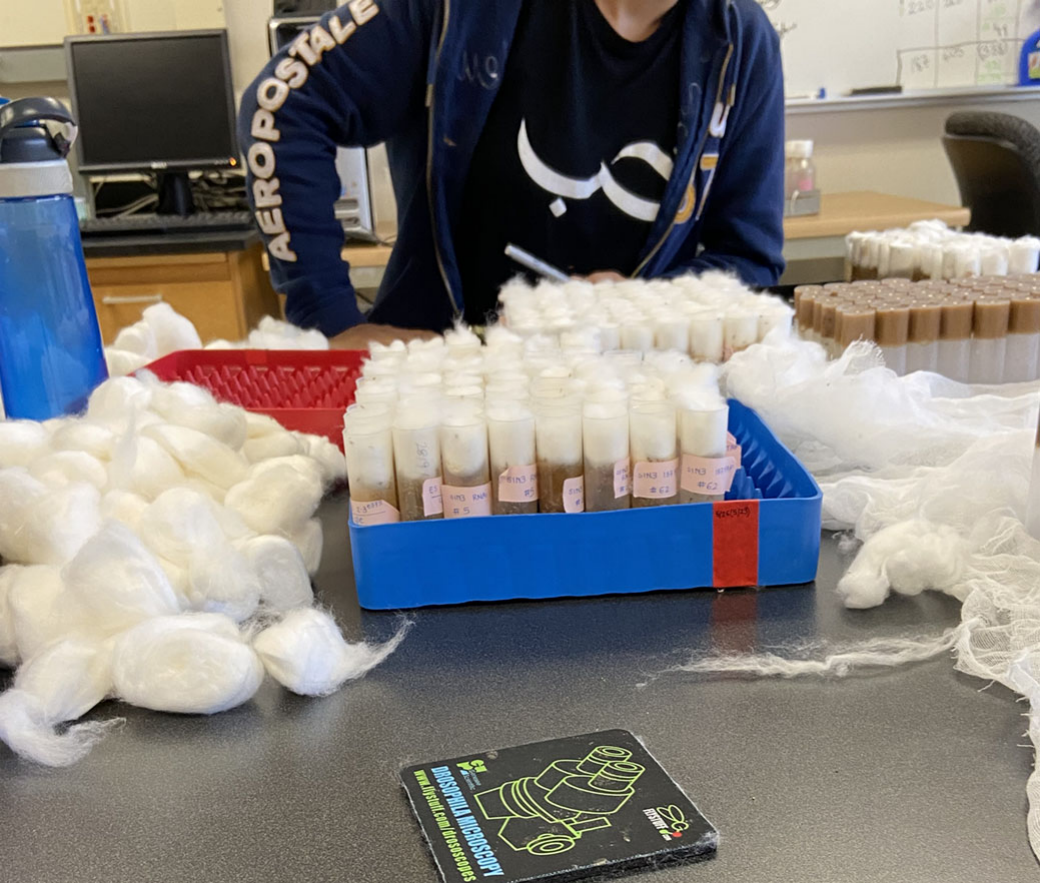
Sitting across from my wife (whom I met in the lab) flipping flies!

**Which part of this research project was the most rewarding?**

I am not a bioinformaticist. The most rewarding aspect of the project was learning how to analyze data. It was difficult teaching myself, but I am proud of what we were able to achieve.

**What do you enjoy most about being an early-career researcher?**

I enjoy the puzzle aspect of the science. As an early-career researcher I have a lot more time and freedom to think about the problems and potential experiments to solve these problems.

**What piece of advice would you give to the next generation of researchers?**

My advice is to find a mentor that you look up to. A good mentor will teach scientific techniques, but also teach you how to be a good scientist and human.

A good mentor will teach scientific techniques, but also teach you how to be a good scientist and human.

**What's next for you?**

I will continue working on interesting problems and learning new techniques that will allow me to find solutions!

## References

[BIO060190C1] Soukar, I., Mitra, A. and Pile, L. (2023). Analysis of the chromatin landscape and RNA polymerase II binding at SIN3-regulated genes. *Biol Open.* 12, bio060026. 10.1242/bio.060026 37850739 PMC10651107

